# Cystic lymphangioma in the peripheral jejunal mesentery in an adult and excision with laparoscopic-assisted surgery: a case report

**DOI:** 10.1186/s12957-019-1713-6

**Published:** 2019-10-24

**Authors:** Hideki Nagano, Toshihisa Kimura, Atsushi Iida, Tamotsu Togawa, Takanori Goi, Yasunori Sato

**Affiliations:** 1 Department of Surgery, National Hospital Organization Tsuruga Medical Center, 33-1, Sakuragaoka, Tsuruga, Fukui 914-0195 Japan; 2 First Department of Surgery, Faculty of Medicine, University of Fukui, 23-3, Matsuokashimoaizuki, Eiheiji-cho, Yoshida-gun, Fukui 910-1193 Japan; 3 Department of Human Pathology Kanazawa University Graduate School of Medicine, 13-1, Takara-machi, Kanazawa, Ishikawa 920-8640 Japan

**Keywords:** Mesenteric lymphangioma, Mesenteric cystic lymphangioma, Jejunum, Laparoscopic-assisted surgery, CD31, Factor VIII-related antigen, D2-40, CD34

## Abstract

**Background:**

Lymphangiomas are uncommon congenital malformations that present mainly in the head, neck, and axillar regions in pediatric patients. Mesenteric cystic lymphangiomas (MCLs), which occasionally present with substantial growth and the invasion of adjacent vital structures, are rarely reported in adults. We report a case of MCL in an adult who was treated with laparoscopic-assisted excision.

**Case presentation:**

A 40-year-old Japanese man visited his family physician for prolonged periumbilical pain. Plain computed tomography (CT) showed a low-density mass in his left abdomen, and he was referred to our hospital 2 weeks later. His abdomen was flat and soft, and no mass was felt upon palpation. Routine laboratory data showed no abnormalities in the blood cell counts. The levels of tumor markers, such as carcinoembryonic antigen (CEA), carbohydrate antigen 19-9 (CA19-9), and cancer antigen 125 (CA125), were within normal ranges. Contrast-enhanced CT was performed, and a low-density mass was observed with an irregular outline and poor contrast, as well as involvement of the peripheral mesenteric artery and partial compression of the adjacent jejunum without dilatation of the oral side of the bowel. The patient was diagnosed with lymphatic cysts and observed for 1 month without symptom exacerbation. Follow-up CT showed no increase in the size of the mass but showed apparent invasion of the jejunal wall without bowel obstruction. Magnetic resonance imaging (MRI) showed intermediate intensity on T1-weighted imaging (T1WI) and high intensity on T2-weighted imaging (T2WI). The coronal view on T2WI clearly showed an accumulation of cystic lesions. We performed tumor excision with partial resection of the jejunum in a laparoscopic-assisted manner. Pathological examination showed multicystic lesions with an attenuated endothelial lining, surrounding rich adipose tissue and scattered smooth muscle fibers; the patient was diagnosed with MCL.

Immunohistochemical assays supported this diagnosis.

**Conclusions:**

This is rare case of MCL presenting in an adult who underwent successful laparoscopic-assisted resection. Mesenteric lymphangioma (ML) should be considered in the differential diagnosis of patients with intraabdominal cysts. Radical excision is optimal, even when the patient is asymptomatic. Laparoscopic-assisted tumor resection is a suitable surgical method for treating MLs located in the peripheral mesentery.

## Background

Mesenteric lymphangiomas (MLs) are known to be uncommon congenital malformations of the lymphatic system [1]; 65% of MLs are present at birth, and 90% of all patients are diagnosed before the age of 2 [2]. Over 95% of lymphangiomas are found in the head, neck, and axillary regions. Isolated small bowel lesions are observed in less than 1% of cases [3] but account for 70% of all intraperitoneal lymphatic tumors [4]. MLs can infiltrate the surrounding organs and cause potentially life-threatening complications, such as traumatic rupture, anemia secondary to intraabdominal or intracavitary bleeding, ischemic tissue necrosis, intestinal gangrene secondary to volvulus, and intermittent intestinal obstruction [5, 6]. MLs occasionally grow and involve main vessels or vital structures and thus become unresectable [7]. In addition, MLs can occur not only in the root of the intestinal mesentery but also in the peripheral part adjacent to the intestinal wall [6]. MLs are traditionally classified into three types: capillary, cavernous, and cystic [8]. Herein, we report a case of mesenteric cystic lymphangioma (MCL) in a 40-year-old man who was successfully treated via laparoscopic-assisted excision.

## Case presentation

A 40-year-old Japanese man experienced periumbilical pain since November 2013, and although the symptoms were mild, they were prolonged. He visited his family physician in January 2014, who noted mild tenderness in the lower left quadrant. The patient underwent an abdominal CT scan. CT revealed a low-density mass that measured 43 × 40 mm in size in the left abdomen. He was referred to the National Hospital Organization Tsuruga Medical Center in February. His past medical and family histories were unremarkable. On examination, the patient had a height of 179.0 cm, a body weight of 98.7 kg, and a body mass index (BMI) of 30.8 and did not present with anemia, icterus, edema, or malnutrition. His abdomen was flat and soft, with mild tenderness in the lower left quadrant on palpation; however, the mass could not be felt.

The laboratory results showed no abnormalities in his blood cell counts; however, an elevation in the serum alanine transaminase (ALT; 60 IU/L), γ-glutamyl transferase (γ-GTP; 108 IU/L), total bilirubin (T-Bil; 1.31 mg/dL), and total cholesterol (T-Chol; 229 mg/dL) levels was observed. The levels of epithelial tumor markers, such as carcinoembryonic antigen (CEA; 2.1 ng/mL), carbohydrate antigen 19-9 (CA19-9; 8.7 U/mL), and cancer antigen 125 (CA125; 11 U/mL), were within normal limits.

**Fig. 1:**
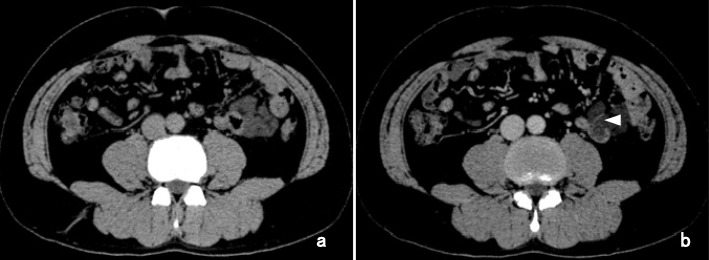
Findings of the CT scan performed in February. **a** A low-density mass showing an irregular outline and measuring 45 × 42 mm in size was detected; the mass was in contact with the jejunum in the left abdomen at the slightly cranial level of the umbilicus, as seen on plain CT. The oral side of the jejunum did not show dilatation. **b** In the contrast-enhanced image, the tumor lacked contrast. The tumor involved the peripheral artery (white arrowhead), indicating that the tumor developed in the mesentery of the jejunum; however, the adjacent jejunum showed good enhancement

The patient underwent a contrast-enhanced CT examination 2 weeks after the CT examination conducted by his family physician because in the previous examination, the patient had not been administered a contrast agent. A low-density mass with an irregular outline and measuring 45 × 42 mm in size was detected in the left abdomen at the slightly cranial level of his umbilicus in the plain phase (Fig. 1a). The tumor was located in the peripheral part of the mesentery of the jejunum and partly compressed the adjacent jejunum. In the enhanced phase, the tumor lacked contrast. The peripheral artery was shown to be involved, indicating that the tumor developed in the mesentery of the jejunum; however, the adjacent jejunum showed good enhancement (Fig. 1b). The oral side of the jejunum did not show dilatation. A lymphatic cyst diagnosis was highly suspected.

**Fig. 2:**
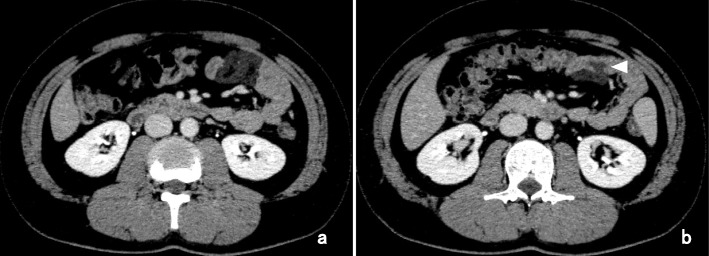
Findings of the CT scan performed in March. **a** The low-density mass showed no increase in size, and the mass did not show contrast in the enhanced phase. Some lower-density components were observed inside of the low-density mass. **b** CT enhancement revealed apparent tumor invasion of the wall of the jejunum (white arrowhead) without ischemia in the adjacent jejunum or dilatation of the oral side of the bowel

**Fig. 3:**
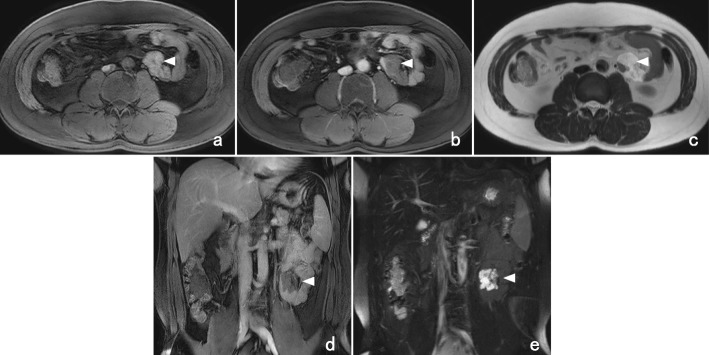
MRI findings. **a** MRI showed the mass with intermediate intensity on T1WI (white arrowhead). **b** The enhanced phase on T1WI showed the mass with a relatively low intensity (white arrowhead). **c** On T2WI, the mass was depicted as a high-intensity tumor (white arrowhead). **d** Coronal view on T1WI. The irregularly shaped mass with a low signal intensity compressed the adjacent jejunum (white arrowhead). **e** Coronal view on T2WI. An accumulation of cystic lesions with a variety of sizes was more clearly depicted (white arrowhead)

The patient’s abdominal symptom passed without exacerbation, and a follow-up examination was conducted in March. One month later, he underwent a scheduled CT examination, and the mass did not appear to have increased in size (Fig. 2a). Enhancement revealed apparent invasion of the wall of the jejunum without ischemia of the adjacent jejunum or dilatation of the oral side of the bowel (Fig. 2b). Magnetic resonance imaging (MRI) showed intermediate intensity on T1-weighted imaging (T1WI) (Fig. 3a) and high intensity on T2-weighted imaging (T2WI) (Fig. 3c). Relatively low intensity was observed with enhancement using gadolinium diethylenetriaminepentaacetic acid (DTPA) contrast medium on T1WI (Fig. 3b). An accumulation of cystic lesions with a variety of sizes was clearly depicted on the coronal view on T2WI (Fig. 3e).

Because it was expected that depiction of the tumor would be made somewhat more difficult by its location and the body shape of the patient, we conducted a sonography examination in reference to the location and properties of the tumor determined by CT and MRI. The lesion showed a low echo; however, a clear image was not obtained.

CT performed by the previous physician and at our institution showed that the tumor was located in the peripheral part of the jejunal mesentery and exhibited slight enteric compression without dilatation of the oral jejunum but no rapid increase in size. The patient hesitated to agree to an immediate operation but did agree to a 1-month observation period. Permeation of the jejunal wall was shown by a second CT examination at our institution performed 1 month later, and from these findings, we highly suspected that the lymphangioma was invading the jejunal wall and thus decided to perform surgery.

We made a preoperative diagnosis of lymphangioma invading the jejunal wall, and with the patient’s informed consent, we performed an operation to relieve his symptoms and obtain a definitive diagnosis of the tumor in April.

**Fig. 4:**
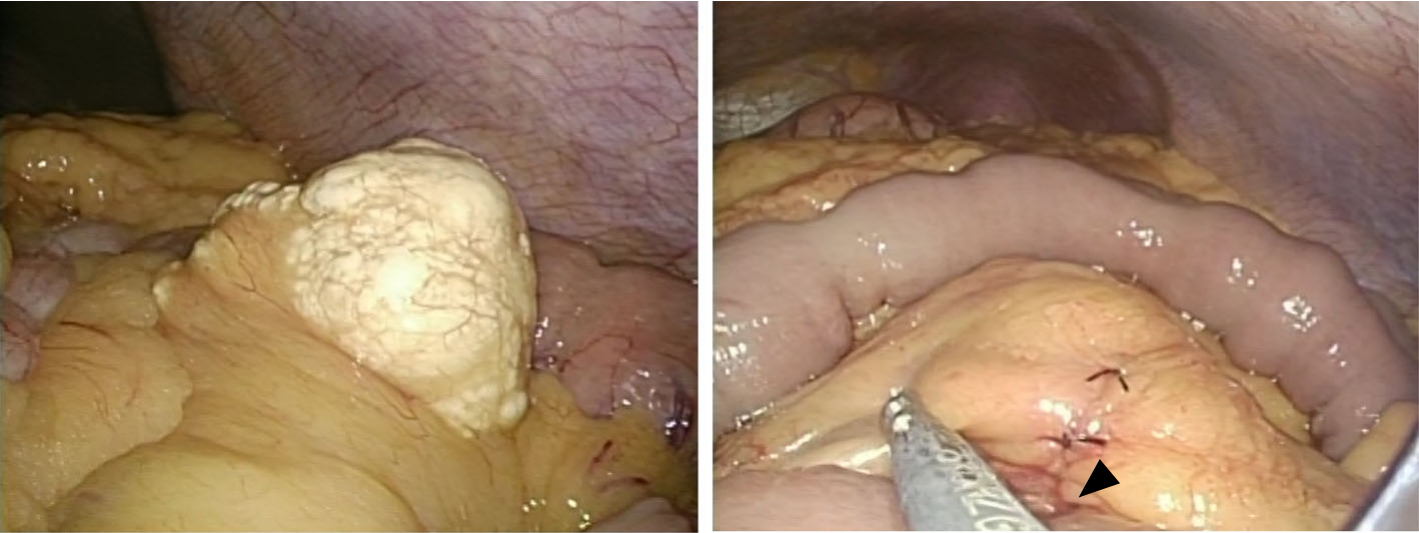
Intraoperative photographs. **a** The omentum was removed from the front of the small intestine, and we found a soft yellowish mass in the mesentery of the jejunum that had invaded the wall of the jejunum. **b** We removed the tumor through the umbilical wound, resected the surrounding mesentery and part of the jejunum, and performed anastomosis in a functional end-to-end manner (arrowhead; anastomotic site)

The patient was placed in the supine position under general anesthesia. Small incisions were made along the superior and inferior border of the umbilicus and connected through the bottom of the umbilicus with an S-shape. The length of the wound was approximately 5 cm. We placed the Lapprotector™ (Model FF0707; Hakko Co., Ltd., Medical Device Division, Japan) in the umbilical wound and then attached the E·Z Access™ device (E·Z Access for FF07; Hakko Co., Ltd., Medical Device Division, Japan). A 12-mm trocar was placed through the E·Z Access device, and pneumoperitoneum was established with carbon dioxide (CO_2_) gas at 8 mmHg of pressure. An accessory trocar (5 mm) was placed on the right side of the 12-mm trocar through the E·Z Access device. Using forceps, we removed the omentum from the small intestinal front and found a soft yellowish mass in the mesentery of the jejunum that had invaded the wall of the jejunum (Fig. 4a). We removed the tumor from the body through the umbilical wound, resected the surrounding mesentery and part of the jejunum, and performed anastomosis in a functional end-to-end manner (Fig. 4b). The total operative duration was 94 min, and the intraoperative blood loss volume was 30 ml.

### Pathological findings

**Fig. 5:**
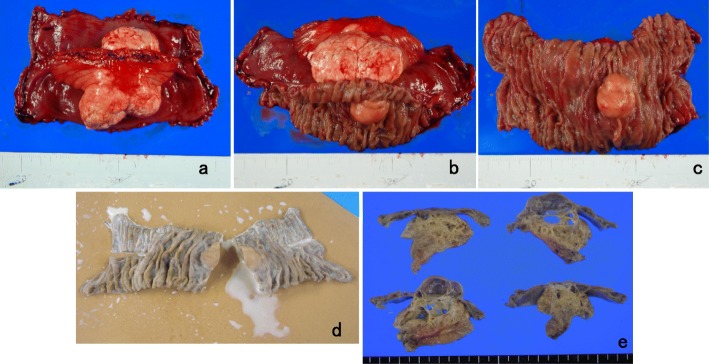
Pathological findings. **a** Macroscopically, the external surface of the mass had a whitish-yellowish and lobulated appearance. The tumor protruded from both sides of the mesenteric leaves was surrounded by the peritoneum of the mesentery, measured 50 × 45 mm in size, and was located in the peripheral part of the mesentery adjacent to the jejunum. **b**, **c** The small intestinal mucosal surface formed a hemispherical uplift approximately 24 × 20 mm in size and was adjacent to the mesenteric lesion without contraction. **d** A formalin-fixed specimen. Café-au-lait-colored chyle-like milky fluid flowed out from inside the tumor after the tumor was cut. **e** The cut surface of the tumor showed multicystic lesions of various sizes without solid components or mural nodularity

**Fig. 6:**
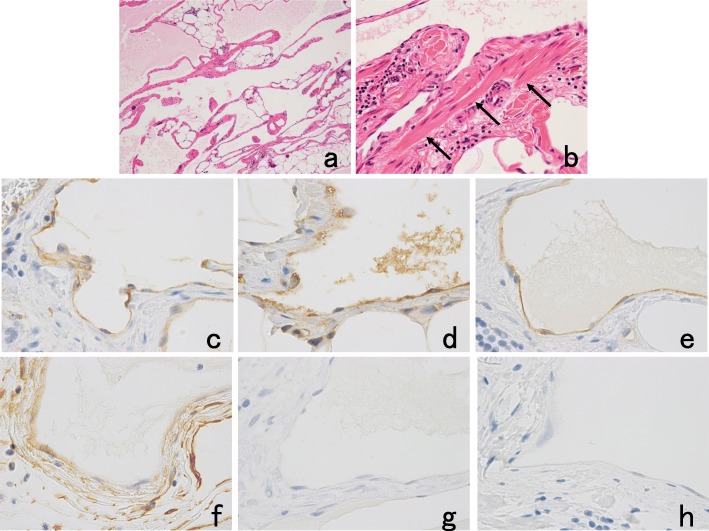
Pathological findings. **a**, **b** Hematoxylin-eosin staining (**a** × 40, **b** × 200). The cyst wall showed an attenuated endothelial lining, surrounding rich adipose tissue and scattered smooth muscle fibers (**b** arrows). Immunohistochemical staining showed that the flat endothelial cells were positive for the endothelial markers CD31 (**c** × 400) and factor VIII-related antigen (**d** × 400), partially positive for the lymphatic endothelial marker D2-40 (**e** × 400) and the undifferentiated pluripotent stem cell marker CD34 (**f** × 400), and negative for cytokeratin (AE1/AE3; **g** × 400) and the mesothelial marker calretinin (**h** × 400)

Macroscopically, the external surface of the mass had a whitish-yellowish and lobulated appearance. The tumor protruded from both sides of the mesentery leaves and was surrounded by the mesenteric peritoneum. The tumor measured 50 × 45 mm in size and was located in the peripheral part of the mesentery and adjacent to the jejunum (Fig. 5a). The small intestinal mucosal surface formed a hemispherical uplift approximately 24 × 20 mm in size adjacent to the mesenteric lesion without contraction (Fig. 5b, c). The cut surface of the mass after fixation by formalin revealed multicystic lesions of various sizes containing a café-au-lait-colored milky fluid similar to chyle (Fig. 5d, e). Histologically, the cyst wall showed an attenuated endothelial lining, surrounding rich adipose tissue and scattered smooth muscle fibers (Fig. 6a, b). In addition, small lymphoid aggregates also appeared focally. Immunohistochemical staining showed that the flat endothelial cells associated with the cysts were positive for the endothelial markers CD31 (Fig. 6c) and factor VIII-related antigen (Fig. 6d), partially positive for the lymphatic endothelial marker D2-40 (Fig. 6e) and the undifferentiated pluripotent stem cell marker CD34 (Fig. 6f), and negative for cytokeratin (AE1/AE3) (Fig. 6g) and the mesothelial marker calretinin (Fig. 6h). Dilated lymphatic ducts were observed in the tumorous lesion of the mesentery and adjacent to this lesion in the muscularis propria and submucosal layer of the small intestine. In the wall of the jejunum without tumor invasion, dilation of the lymphatic ducts was not observed. These findings were consistent with cystic lymphangioma.

The patient recovered successfully and was discharged from the hospital 9 days after the operation.

## Discussion

Lymphangiomas are congenital malformations of the lymphatic vessels and are usually associated with pediatric disease [9]. Sabin [10] divided the development of the lymphatic system into two stages: (1) the development of isolated lymph sacs derived from veins that are united by the thoracic duct and (2) the peripheral growth of lymph vessels that sprout from the endothelial lining of the sacs. The lymph sacs are transformed into the plexus or lymphatic capillaries by bridging the lumen with bands of connective tissue from which chains of lymph nodes are derived. Sabin [11] also reported that the lymphatic vessels were developed from the following five primordia: (1) paired jugular sacs lateral to the jugular veins, (2) an unpaired retroperitoneal sac at the mesenteric root, and (3) paired posterior sacs related to the sciatic veins (centrifugal theory). Godart [12] stated that the etiology of lymphangioma is related to abnormal development of the lymphatic system due to communication failure in a branch or branches within the central system, thus explaining why cystic lymphangiomas are found in the same position as fetal lymph sacs (centripetal theory). Approximately 95% of lymphangiomas are found in the head, neck, and axilla, and the other 5% occur in the mediastinum and abdominal cavity, including the mesentery, retroperitoneum, and bones [9]. It is presumed that there is a high association between developing lymphatic vessels and cystic lymphangioma. MLs account for less than 1% of all lymphangiomas [3]. Abdominal lymphangiomas have been reported in the mesentery, accounting for more than half of the cases (58/107), followed by the retroperitoneal space (11/107), spleen (9/107), colon (8/107), small intestine (8/107), pancreas (5/107), and other locations [13]. MLs result from a lack of communication between the small bowel lymphatic tissue and the main lymphatic vessels during fetal development, resulting in blind cystic lymphatic spaces lined by endothelial layers [14]. However, some lymphangiomas diagnosed in adults were assumed to be acquired and to have developed through different causes and pathways, such as obstruction of the existing lymphatic channels by an inflammatory fibrotic process, surgery, radiation exposure, abdominal trauma, or lymphatic hamartoma [1, 15–17]. Some reports have described no mesenteric masses in previous abdominal surgeries [16, 18]. Although benign in nature, MLs may cause significant morbidity or mortality due to their large size and critical location. MLs can infiltrate the surrounding organs and cause potentially life-threatening complications, such as traumatic rupture, anemia secondary to intraabdominal or intracavitary bleeding, ischemic tissue necrosis, intestinal gangrene secondary to volvulus, and intermittent intestinal obstruction [1]. One report described an ML lesion that encased the whole superior mesenteric artery [7]. MLs that occur in a relatively peripheral part of the mesentery, such as in our case, seemed to be diagnosed at a relatively early stage due to compression or invasion of the adjacent small bowel resulting in symptoms. The most common presentation is a freely mobile and nontender abdominal mass with partial small intestinal obstruction [7, 19], and tumor bleeding has rarely been reported [20]. However, this type of tumor could have a tendency to cause intestinal volvulus [9, 18, 21, 22].

Lymphangiomas are traditionally classified into three histological types: capillary (simple), cavernous, and cystic [8]. The capillary (simple) type usually originates in the skin and contains uniform, small, thin-walled lymphatic spaces. The cavernous type is composed of dilated lymphatic spaces of various sizes, is associated with lymphoid stroma, and generally maintains a connection with the adjacent normal lymphatic spaces. The cystic type consists of dilated lymphatic spaces of various sizes associated with collagen and smooth muscle bundles in the stroma but lacks connection to the adjacent normal lymphatic spaces [4, 23].

Differential diagnoses include a wide range of cystic intraabdominal lesions, such as mesenteric cysts, abdominal lymphomas, secondary metastases from an unknown primary tumor, tuberculosis, hydatid disease, small bowel adenocarcinomas, and rare mesenteric tumors, including desmoid tumors, schwannomas, smooth muscle tumors, sarcomas, cystic mesotheliomas, lymphangiosarcomas, and lymphangiomas with myxoid degeneration [17, 24].

To prove the lymphatic origin of the endothelial cells of cysts, we performed immunohistochemical analysis using a representative set of markers consisting of CD31, factor VIII-related antigen, D2-40, CD34, AE1/AE3, and calretinin for the diagnosis of lymphangioma [25]. Immunohistochemical staining of samples from the presented case showed that the flat endothelial cells associated with the cysts were positive for CD31 and factor VIII-related antigen, partially positive for D2-40 and CD34, and negative for cytokeratin (AE1/AE3) and calretinin. Factor VIII-related antigen, CD31 and CD34 can provide clues for the diagnosis of lymphangioma; however, both CD31 and CD34 label blood vessels and lymphatic endothelial tissue, making it difficult to differentiate lymphangioma from angioma [26]. D2-40 is a monoclonal antibody to the transmembrane mucoprotein, which is expressed by lymphatic endothelial cells, among others [26]. D2-40 shows immunoreactivity to only lymphatic endothelial tissue, so it is a marker specific for tissues of lymphatic origin [27]. Calretinin is a representative mesothelial marker, and reactivity to this marker can be used to distinguish disorders with a mesothelial cell origin, such as benign multicystic mesothelioma [23].

To treat ML, radical surgical excision is recommended, even when patients are asymptomatic, because ML can grow rapidly and invade adjacent structures, resulting in complications and the risk of sarcoma transformation upon irradiation [4]. However, radical excision can sometimes be technically impossible [4]. The recurrence rate has been reported to be 10% due to difficulty in resecting the entire cyst wall; the excision of retroperitoneal cysts is especially challenging due to their close proximity to vital retroperitoneal structures, making resection hazardous or even impossible [28, 29].

Minimally invasive surgery is a surgical treatment option. A relatively small ML, especially one that develops in the peripheral area of the mesentery, as in our case, is a good indication for laparoscopic-assisted surgery because the peripheral ML lesion can be easily removed from the body through a minimal wound and resection, and anastomosis can be performed under direct observation. A precise preoperative evaluation of the diagnostic images to determine the size, anatomical location, extent, and involved structures as well as the predicted laparoscopic surgical field aids in identifying an appropriate port site and wound length. Losanoff et al. [17] noted four types of MCLs: type 1, pedicled MCLs; type 2, sessile MCLs located in the mesenteric boundary; type 3, MCLs with retroperitoneal extension; and type 4, multicentric MCLs. MCLs located in the peripheral part of the mesentery were not included in this classification system. Kim et al. [6] presented a modified classification system for MLs consisting of four groups: group 1, MLs involving the intestinal wall; group 2, pedicled MLs with no relationship to the mesenteric vessels; group 3, sessile MLs located in the mesenteric boundaries near the mesenteric vessels; and group 4, multicentric and diffusely infiltrated MLs. The lesion in our case was classified into group 1. Among the 25 cases in Kim’s report, 14 were classified into group 1, and all of the lesions were excised with segmental resection of the intestine. The peripheral type of ML did not seem to be a rare type. However, there were no descriptions regarding the size of each tumor, and the number of cases limited to the peripheral part of the intestinal mesentery, apart from the root of the mesentery or main mesenteric vessels, was unknown. Of 14 patients, two patients in group 1 underwent laparoscopic-assisted surgery. This classification system is useful for identifying the peripheral ML type as well as involvement of the intestinal wall, and considering the volume of the tumor, it could contribute to evaluating the indication for laparoscopic-assisted surgery.

We used a pressure of 8 mmHg for pneumoperitoneum during the operation in this case. In our faculties, laparoscopic surgery begins with pneumoperitoneum at a pressure of 8 mmHg to avoid referred pain by diaphragm hyperextension, symptoms of high carbon dioxide in the blood, atelectasis, and phlebothrombosis, and if a good visual field cannot be maintained, we increase the pneumoperitoneal pressure incrementally. Fortunately, we were able to accomplish the intended procedure with a sufficient surgical field of view at 8 mmHg of pneumoperitoneal pressure in this case. However, if necessary, the pneumoperitoneal pressure can be increased to 12 or 15 mmHg to provide a better surgical field of view.

The Lapprotector™ and E·Z Access™ devices are useful for laparoscopic-assisted tumor resection because they allow the use of multiple ports with minimal trauma and can also be used in single-port laparoscopic surgery (SPLS). We used two ports (one scope port and one forceps port) to remove the omentum. It seems that the excision of mesenteric tumors with a portion of the small intestine using a laparoscopic technique provides anatomical ease and the advantages of minimal surgical invasiveness, a reduced wound number and length, less postoperative pain, fewer wound-related complications, and improved cosmetic outcomes [30–32].

## Conclusions

We report a case of MCL in an adult patient who was successfully treated by laparoscopic-assisted excision. ML should be considered in the differential diagnosis of patients with intraabdominal cystic masses. The optimal treatment is radical excision, even when the patient is asymptomatic. Laparoscopic-assisted tumor resection is a suitable surgical method for treating MLs located in the peripheral part of the mesentery.

## Data Availability

All data generated or analyzed during this study are included in this published article.

## Abbreviations

ALT: Alanine transaminase
BMI: Body mass index
CA125: Cancer antigen 125
CA19-9: Carbohydrate antigen 19-9
CD31: Cluster of differentiation 31
CD34: Cluster of differentiation 34
CEA: Carcinoembryonic antigen
CO_2_: Carbon dioxide
CT: Computed tomography
Gadolinium DTPA: Gadolinium diethylenetriaminepentaacetic acid
MCL: Mesenteric cystic lymphangioma
ML: Mesenteric lymphangioma
MRI: Magnetic resonance imaging
SPLS: Single-port laparoscopic surgery
T1WI: T1-weighted imaging
T2WI: T2-weighted imaging
T-Bil: Total bilirubin
T-Chol: Total cholesterol
γ-GTP: γ-Glutamyl transferase
